# From commitment to implementation: lessons learnt from the first National Strategy for the Reduction of Teenage Pregnancy in Sierra Leone

**DOI:** 10.1080/26410397.2020.1818376

**Published:** 2020-10-19

**Authors:** Regina Bash-Taqi, Katherine Watson, Elsie Akwara, Emmanuel Adebayo, Venkatraman Chandra-Mouli

**Affiliations:** a Director, Institute for Development (IfD), Freetown, Sierra Leone; b Freelance Consultant, Singapore; c Consultant, Department of Sexual and Reproductive Health and Research/Human Reproduction Programme, World Health Organization, Geneva, Switzerland; d Research Fellow, Institute of Child Health, College of Medicine, University of Ibadan, Ibadan, Nigeria; e Scientist, Department of Sexual and Reproductive Health and Research/Human Reproduction Programme, World Health Organization, Geneva, Switzerland

**Keywords:** policy-making, political prioritization, reproductive health, young people, adolescent pregnancy, Ebola

## Abstract

This study provides insight into the extent to which public commitment to reduce teenage pregnancy made by the President of Sierra Leone made the issue a political priority and the factors that facilitated and hindered this. Using historical observations from government and civil society actors who were involved in the formulation and implementation of the country’s National Strategy for the Reduction of Teenage Pregnancy (NSRTP), the study presents lessons learnt, with a particular focus on advocacy. It does not examine the extent to which the NSRTP was operationalised and its objectives fulfilled. Findings indicate that the availability of locally relevant data as well as advocacy from international and national NGOs were factors that led to the President’s commitment and the development of a national strategy. Whilst continued verbal support from political leaders and administrative mechanisms for implementation assured that teenage pregnancy reduction stayed on the political agenda, the scarcity of resources as well as the necessary diversion of efforts and resources to the Ebola epidemic impeded implementation. Overall, the findings demonstrate that public commitments made by political leaders – starting with President Ernest Bai Koroma’s public declaration in 2012 – kick-started efforts to reduce teenage pregnancy in Sierra Leone; and that despite inadequate human and financial resources for the implementation of the NSRTP, actions taken by both the government and partners over time have contributed to tangible progress.

## Résumé

Cette étude montre dans quelle mesure l’engagement public du Président sierra-léonin de réduire les grossesses chez les adolescentes a fait de la question une priorité politique et elle décrit les facteurs qui ont facilité ou contrarié le processus. Se servant d’observations historiques d’acteurs du Gouvernement et de la société civile qui ont participé à la formulation et la mise en œuvre de la stratégie nationale du pays pour la réduction des grossesses chez les adolescentes (NSRTP), l’étude présente les leçons apprises, en se centrant particulièrement sur le plaidoyer. Elle ne se demande pas si la NSRTP a été opérationnalisée et si elle a atteint ses objectifs. Les conclusions indiquent que la disponibilité de données pertinentes pour le niveau local ainsi que les activités de plaidoyer d’ONG nationales et internationales ont été des facteurs qui ont abouti à l’engagement du Président et à la mise au point d’une stratégie nationale. Si la poursuite du soutien oral des dirigeants politiques et des mécanismes administratifs à la mise en œuvre a permis de maintenir la réduction des grossesses chez les adolescentes à l’ordre du jour politique, la rareté des ressources ainsi que la nécessaire réorientation des activités et des ressources au profit de l’épidémie d’Ebola ont contrarié la mise en œuvre. Dans l’ensemble, les résultats démontrent que les engagements publics des dirigeants politiques, à commencer par la déclaration publique du Président Ernest Bai Koroma en 2012, ont initié les efforts pour réduire les grossesses d’adolescentes en Sierra Leone; et qu’en dépit de l’insuffisance des ressources financières et humaines pour l’application de la stratégie nationale, les mesures prises aussi bien par le Gouvernement que par ses partenaires au fil du temps ont contribué à des progrès tangibles.

## Resumen

Este estudio analiza en qué medida el compromiso público adquirido por el presidente de Sierra Leona para disminuir el embarazo en la adolescencia otorgó prioridad política a este asunto, y examina los factores que facilitaron u obstaculizaron este objetivo. Utilizando observaciones históricas de actores del gobierno y la sociedad civil que participaron en la formulación e implementación de la Estrategia Nacional para la Reducción del Embarazo en la Adolescencia (NSRTP, por sus siglas en inglés), el estudio presenta lecciones aprendidas con énfasis en promoción y defensa. No examina en qué medida se operacionalizó la NSRTP y se cumplieron sus objetivos. Los hallazgos indican que la disponibilidad de datos pertinentes a nivel local, así como actividades de promoción y defensa realizadas por ONG internacionales y nacionales, fueron factores que propiciaron el compromiso del Presidente y la formulación de una estrategia nacional. Aunque el continuo apoyo verbal de líderes políticos y mecanismos administrativos de implementación garantizaron que la reducción del embarazo en la adolescencia permaneciera en la agenda política, la escasez de recursos, así como el necesario desvío de esfuerzos y recursos a la epidemia de Ébola, bloquearon la implementación. En general, los hallazgos demuestran que los compromisos públicos adquiridos por líderes políticos, empezando con la declaración pública del presidente Ernest Bai Koroma en el año 2012, pusieron en marcha los esfuerzos por reducir el embarazo en la adolescencia en Sierra Leona; y que, a pesar de escasos recursos humanos y financieros para la implementación de la NSRTP, las acciones tomadas por el gobierno y socios a lo largo del tiempo han contribuido a progresos tangibles.

## Introduction

Evidence from the past three decades demonstrates that public commitments can be important starting points for achieving sexual and reproductive health (SRH) and development goals. The Millennium Development Goals (MDGs), for example, were successful in generating both political and financial support for initiatives across the globe, as evidenced by the significant progress made towards some health and development goals between 2000 and 2015.^1^ At the regional level, the Maputo Plan of Action is an example of an agreement intended to spur countries into action in Africa for the advancement of sexual and reproductive health and rights (SRHR). Within the first five years, a number of countries in the region developed roadmaps for maternal and newborn health, made significant progress in scaling up linkages between SRH and HIV, passed new laws to protect women against violence, criminalised harmful practices against women, and expanded family planning programming.^2^ At the national level, political commitment to teenage pregnancy reduction has been shown to be a critical element to the success of effective large scale programmes in contexts as diverse as Chile, Ethiopia and England.^3^ More experiential learning is needed to understand the factors that influence whether and how such political commitments translate into meaningful action for the advancement of SRH.

Knowing that policy-makers are “burdened with thousands of issues and have limited resources to deal with them”,^4^ important lessons can be drawn from an understanding of how certain development issues get placed on the agenda of political leaders and, once there, how they remain. Schiffman sets out a useful framework for understanding political prioritisation; although used in the context of maternal health in low- and middle-income countries, his three criteria prove useful for reviewing the political prioritisation of other health and development goals, including teenage pregnancy. According to Schiffman, a political priority is present when: (1) national political leaders publicly and privately express sustained concern for the issue; (2) government, through an authoritative decision-making process, enacts policies that offer widely embraced strategies to address the problem; and (3) government allocates and releases public budgets commensurate with the problem’s gravity.^4^

This research study seeks to understand when and how teenage pregnancy reduction was placed on the political agenda of Sierra Leone; the extent to which teenage pregnancy reduction became a political priority during the formulation and implementation of the first National Strategy for the Reduction of Teenage Pregnancy (NSRTP); and which factors influenced the process of political prioritisation. Using historical observations from government and civil society actors who were involved in the formulation and implementation of the NSRTP, this research study draws out lessons learnt. However, the paper does not comment on the extent to which the NSRTP was implemented and succeeded in achieving its objectives. Schiffman’s framework for assessing political prioritisation was influential in shaping the research questions and in providing the analytic basis for the second research question; however, the process-tracing methodology that he used in relation to maternal health in his 2007 paper was not followed. The following research questions were used to guide the evidence collection, data analysis and organisation of results:

1. What factors contributed to the President of Sierra Leone making teenage pregnancy reduction a national priority in 2012?
2. To what extent did teenage pregnancy reduction become a national priority following the President’s declaration?
3. What factors facilitated or obstructed teenage pregnancy reduction remaining a political priority in Sierra Leone during the implementation of the first NSRTP (2013–2015)?

## Background

Sierra Leone is located on the west coast of Africa bordering Guinea and Liberia and has a total population of seven million, 22% of which are adolescents (aged 10–19).^5^ It is an economy driven largely by the agricultural industry, and 57.9% of the total households in the country are agricultural households.^5^ It is estimated that more than half of the population lives in poverty.^6^ After gaining independence in 1961, the country experienced internal conflicts, culminating in several attempted coups and a decade long civil war ending in 2002. Although the country experienced economic progress in the years following the war, this was interrupted by the Ebola Virus Disease outbreak in 2014, which had a substantial impact on development in all areas.

With regard to health, Sierra Leone ranked very low in many domains after emerging from the civil war. The country was recorded as having had the world’s highest under-five mortality rate in 2012, the third highest maternal mortality ratio in 2010, and the twenty-third highest adolescent fertility rate in 2011.^7^ In the years immediately following the civil war, there were limited efforts to address adolescent SRH and, as stated by the government itself, a “paucity of strategic direction” in this area.^8^ At that time, 34% of all pregnancies occurred amongst teenage girls, and the adolescent fertility rate stood at 122 per 1000; further, an estimated 40% of all maternal deaths were among adolescents.^9^ Also revealing were the data for indicators relating to early marriage and access to contraception; the introductory text to the Government of Sierra Leone’s NSRTP revealed that 50% of girls were married or in consensual union before the age of 18, whilst 94.7% of young women aged 15–19 who were married or living with a partner were not using any contraceptive method.^8^ Further, high rates of early marriage, low contraceptive prevalence amongst adolescents, poverty, sexual abuse, social norms and transactional sex were all identified in the NSRTP as factors contributing to the high rate of teenage pregnancy in Sierra Leone.^8^

## Methods

Data presented in this paper were collected through a literature review and key informant interviews. The literature search was conducted using Google Scholar, PubMed, Cochrane Database and MEDLINE with the following key words and phrases: *teenage pregnancy and Sierra Leone*; *political priority and adolescent health*; *Sierra Leone teenage pregnancy strategy*; *policies, adolescents and Sierra Leone*. Bibliographies included in selected articles were also scanned for other relevant articles, and key experts and researchers were asked to submit relevant documentation. To be included, the literature needed only to cover the time period just before or during the implementation of the NSRTP in Sierra Leone (2010–2015). A template that drew out the relevant findings for each of the three research questions was utilised to ensure a consistent approach between the two authors conducting the literature review. Additionally, ten policy documents from Sierra Leone that relate to young people’s health and/or SRH, including the NSRTP, were consulted.

Once the literature review was completed, a standardised semi-structured interview guide was developed based around the three research questions. In relation to the second research question on national priority, interviewees were asked about the extent to which the NSRTP led to increased and sustained public discussion on teenage pregnancy, as well as budget/investment and policy/programmatic action. The interviews were conducted by an experienced researcher in Sierra Leone affiliated with the Institute for Development (IfD); these were completed within a two-week period in spring 2019. All interviews were conducted in English and were recorded with the consent of the key informants. From the recordings, comprehensive notes were made in a data analysis template specifically developed for this study.

Interviews were conducted with 11 key informants who were directly involved in the development or implementation of the NSRTP. Amongst the key informants were six government officials, three local NGO workers and two international NGO representatives. Key informants were selected using the team’s knowledge of individuals and organisations that had been actively involved in teenage pregnancy prevention work in Sierra Leone during the time period leading up to the launch of the NSRTP and its application. Minutes from national strategy development meetings were consulted to ensure that a comprehensive initial list of key informants was drawn up. From an initial list of 21 possible key informants, 11 were selected for interview, ensuring representation from government, NGOs and UN agencies. After conducting eight interviews, the researcher reached saturation point on all of the research questions; despite this, the final three interviews were completed.

Ethical considerations included ensuring participants were fully informed of the purpose of the study, how the data would be used and of their right to withdraw from the study at any time. All participants signed consent forms and were assured of confidentiality and anonymity. The authors also received an ethics waiver from the Sierra Leone Ethics and Scientific Review Committee as the article is a retrospective review of data collected by one of the first authors (Bash-Taqi) during a consultancy assignment with the Ministry of Health and Sanitation. The consultancy agreement permitted the author to utilise data collected for academic purposes.

Analysis of the interview data was done through thematic coding. A template structured around the three research questions and, within the second research question, Schiffman’s three criteria for assessing political prioritisation. Data were entered into the template from the interviews and thematic coding was used to draw out the findings. The findings from the interviews and literature were reviewed and discussed amongst the authors both before and during the drafting of the manuscript.

### Literature reviewed

A total of 21 documents – 5 from peer-reviewed journals and 16 from the grey literature – were found through the searches and bibliographic reviews (Table 1). All of the documents were reviewed, though only two of the peer-reviewed journal articles (Bruce, Elston) contained information relevant to the research questions, whilst most of the grey literature reviewed was relevant. The ten policy documents consulted are shown in Table 2.

**Table 1.** Literature review summary

Article reviewed	Type	Objective	Study/evaluation design
*Akara^10^	Peer-reviewed	To highlight experiences of girls participating in empowerment and leadership programmes	Narrative
Bash-Taqi^11^	Grey literature	To describe the level of readiness to implement the Global Strategy for Women, Children and Adolescent health (2016–2030) in Sierra Leone	Epidemiological review and situational analysis based on 11 semi-structured key informant interviews of purposively selected policy-makers and a snowball of other key actors
Bransky et al^2^	Grey literature	To understand the lived experiences of child marriage in order to create a more holistic picture of life for married and pregnant girls and those at risk of marriage and teenage pregnancy	Cultural theory analysis based on semi-structured discussion groups and individual ethnographic interviews
Bruce^12^	Peer-reviewed	To respond to questions from the Editor of the Journal of Virus Eradication	Viewpoint response on the importance of fulfilling the basic human rights of adolescent girls and their relationship with viral epidemics such as HIV
Coinco^13^	Grey literature	To determine the present community practices and beliefs surrounding teenage pregnancy and teenage motherhood and the communities’ existing responses; to establish the factors leading to teenage pregnancy relating to sexual behaviour of girls, boys and men, reproductive health knowledge, power relations, peer pressure, and others; to establish the impact of teenage pregnancy and teenage motherhood on their education and mental health; and to provide recommendations on how to address the problem of teenage pregnancy and improve the lives of teenage mothers	Multi-method study with both qualitative and quantitative data sources, including key informant interviews, focus group discussions, in-depth interviews, positive deviance approach and questionnaires
De Koning et al^14^	Grey literature	To provide insights on girls’ decision-making around sex, pregnancy and marriage, as well as the resulting consequences for girls’ choices	Exploratory and descriptive study involving case study development, in-depth interviews, key informant interviews and focus group discussions
Denney et al^15^	Grey literature	To map out the scope of the problem of teenage pregnancy in Sierra Leone in the post-Ebola context by providing an overview of common intervention types, gaps and current programming responses	Semi-structured interviews with government and donor agencies and local and international NGOs
*Denney et al^16^	Grey literature	To provide a broad set of reflections on current programming approaches – what is missing, what some of the challenges of implementation are, and whether the underlying logic implicit in programme approaches makes sense	Qualitative study involving interviews and focus group discussions a variety of stakeholders
Diarra et al^17^	Grey literature	To determine the factors that lead to teenage pregnancy and to identify the consequences of teenage pregnancy in Sierra Leone	Mixed-method study involving quantitative data from the 2008 SLDHS and focus group discussions
*Dunlop and Penzhorn^18^	Grey literature	To comment on teenage pregnancy, social support and livelihood	Commentary
Ebola Deeply^19^	Grey literature	–	Interview with Chernor Bah, Founder of Purposeful
Elston et al^20^	Peer-reviewed	To identify and quantify the impact of the outbreak on population health and health systems	Mixed-method study involving interviews, focus groups, and interrogation and analysis of data from health facilities, district health records and burial teams; *T*-tests performed to compare periods before and during the outbreak
*Farzaneh^21^	Grey literature	To examine the root causes of teenage pregnancy	Implementation of pilot projects in 7 districts with the aim of reducing the prevalence of teenage pregnancy in each; focus groups discussions and key informant interviews used to assess change
Kosia^22^	Grey literature	To examine the “cultural dynamic” of teenage pregnancy in Sierra Leone and analyse the teenage pregnancy reduction strategic plan to deduce whether the plan may be successful in achieving its goal	Data drawn from a four-month (January–April 2014) practicum experience with the Ministry of Health and Sanitation in Sierra Leone, complemented by literature searches on select websites relating to the NSRTP
*Republic of Sierra Leone^23^	Grey literature	To analyse the risk factors contributing to teenage pregnancy among adolescent girls who became pregnant during the Ebola outbreak.	Mixed-methods study involving questionnaire and semi-structured interviews
*Restless Development^24^	Grey literature	To reflect the views of young people across Sierra Leone with respect to the key issues affecting their lives	Study involving focus group discussions, in-depth interviews and literature review
*Runsewe-Abiodum and Bondi^25^	Peer-reviewed	To describe the health seeking habits of a cohort of teenage mothers attending an under-five clinic with a view to determining the impact on the survival of their children	Descriptive cross-sectional and prospective study
Shirley et al^26^	Grey literature	To assess the impact of the community-driven interventions on the incidence of teenage pregnancy and on a range of immediate and medium-term outcomes, including knowledge levels, access to/use of contraception and social norms	Baseline study that formed part of a larger quasi-experimental action research initiative with multiple phases and randomised cluster trial design
*Stark et al^27^	Grey literature	To test the value of non-formal-formal linkages and the effectiveness of community owned and driven interventions that seek to reduce teenage pregnancy	Quasi-experimental study with randomised trial design that included baseline and endline surveys
Stark et al^7^	Peer-reviewed	To evaluate associations between living arrangement and orphanhood on recent sexual activity and pregnancies out of wedlock in Sierra Leone	Study involving surveys in two rural districts with 530 adolescents
*Steifel^28^	Grey literature	To understand how the Ebola epidemic impacted maternal and child health in Liberia and Sierra Leone.	Documentation

*Articles not cited in results.

**Table 2.** National policies and strategies Sierra Leone

1926	Prevention of Cruelty to Children Act, cap 31
1993	National Health Policy (reviewed in 2002)
2003	National Youth Policy
2007	Child Rights Act
2010	Free Healthcare Initiative
2010	First National Health Sector Strategic Plan [NHSSP] (2010–2015)
2011	Reproductive, Newborn and Child Health Strategy (2011–2015)
2013	Let girls be girls, not mothers: NSRTP (2013–2015).
2016	National Reproductive, Maternal, Newborn, Child and Adolescent Policy (2017–2021)
2017	National Strategy for the Reduction of Adolescent Pregnancy and Child Marriage (2018–2022)

## Results

Ernest Bai Koroma became President of Sierra Leone in 2007. Whilst the pressure on the government to address teenage pregnancy had been building for years, some are able to pinpoint the exact moment when the President decided to act. Bash-Taqi’s review quotes a UN staff member as saying that it was upon hearing that one in every three girls was pregnant before the age of eighteen during a National AIDS Secretariat (NAS) meeting that teenage pregnancy reduction became a top priority for the President:

“ … [S]omeone mentioned at the meeting that the strategies that were aimed at addressing HIV/AIDS should be (in) synergy with activities addressing teenage pregnancy and I think it was a little by chance that the person who made that intervention just simply quoted from the latest DHS that one in every three girls in Sierra Leone was pregnant before 18 and when the President heard that … he actually stopped the meeting and asked [for] confirmation of data … he turned to his Health Advisor at the time and said that this was a major issue that he wanted to see addressed and asked the Health Advisor to take the action point. … .”^11^ *(p. 34)*

**Figure 1.**
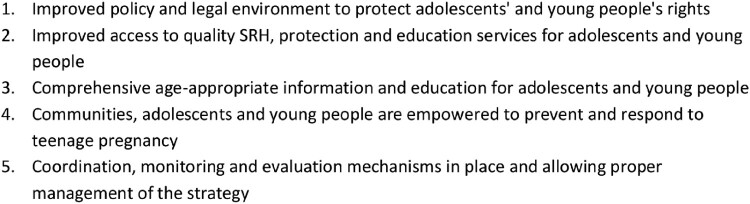
Expected outputs of NSRTP (2013–2015)

Based also on interviews with key informants, this study considers this “lightbulb” moment to be what some refer to as the President’s “declaration” on teenage pregnancy. It was after this moment that the development of the first national strategy began. The stated outcome of the strategy was “*to have reduced the adolescent fertility rate from 122/1000 to 110/1000*” by 2015; furthermore, it was decided that the number of “*girls who had given birth before 19 will be reduced from 34% to 30%’ during the same timeframe*”.^8^ Additionally, there were five expected outputs of the NSRTP (Figure 1).

The following sections address the authors’ attempts to understand, firstly, the contextual factors that contributed, or led to, the President’s “declaration”; in other words, to understand how teenage pregnancy made its way onto the political agenda. The second section presents findings related to the second research question – namely, whether and to what extent teenage pregnancy remained a political priority following this “declaration”. In the third section the factors that were found to facilitate and obstruct teenage pregnancy becoming and remaining a political priority are explained.

### What factors contributed to the President of Sierra Leone making teenage pregnancy reduction a national priority in 2012?

This study identified two important factors that led to the President’s declaration on teenage pregnancy reduction. The first was the availability of impactful, locally relevant data relating to teenage pregnancy in Sierra Leone; these data not only presented the up-to-date status of adolescent fertility and health but, also, the linkages of adolescent pregnancy with other issues of concern, including poverty, education of girls and transactional sex. The second factor was advocacy from international and national NGOs focused on the Government of Sierra Leone (GoSL) to take action.

In the years prior to the presidential declaration, there was an increase in the availability of quality, locally relevant data and evidence relating to teenage pregnancy, early marriage and access to contraception. Key informants emphasised the importance of the availability of these and other data in elevating teenage pregnancy to the level of public consciousness and, eventually, political importance: “ … *[From the reports] we realized that girls as young as 9 [years old] were getting pregnant and were having children*” (Representative, UN agency).

To complement the statistics, reports such as those published by UNICEF and UNFPA highlighted the scale of the problem and the seriousness of the impact that teenage pregnancy was having on girls’ lives.^13–15,17^ One nationwide assessment, for example, reported on the high proportion of maternal deaths as well as the high percentage of school dropouts resulting from teenage pregnancy.^13^ Not only did such reports highlight the impact of pregnancy on girls’ health outcomes, but they made clear the linkages with poverty reduction, an issue high on the president’s agenda.^2^ When asked which factors brought teenage pregnancy to the President’s attention, several key informants responded with reference to its links with poverty reduction:

“ … if you have so many of such cases [teenage pregnancy] country wide [in] Sierra Leone [that is] deeply entrenched in poverty, the anti-poverty strategy and the anti-poverty efforts of the government and the international community will sum up to zero because the underlying factors of poverty will be so strong and they will be very difficult to eradicate. … . In my opinion, one of the factors I thought the president realized [is] that when the child becomes a mother, the child will not be able to take care of the baby and that will further expose the baby and the mother into the vicious cycle of poverty.” *(Representative, National Secretariat for the Reduction of Teenage Pregnancy)*

“ … [girls] dropped out of school and the tendency for them to return to school will be limited. We have less and less families coming out of the poverty cycle because most of the families were very vulnerable and so were the children.” *(Representative, UN agency)*

In the context of all the new, credible research on the magnitude and consequences of teenage pregnancy, the advocacy from UN agencies on the GoSL to act mounted. Indeed, UNICEF, UNFPA and WHO had long been pushing for a national strategy addressing the high teenage pregnancy rates in the country. A UNICEF report published in 2010, for example, called for the government to promote open discussion on SRH and to commit for the long-term.^19^ A UNFPA-sponsored study published a year later entitled “Children bearing children” called attention to the government’s lack of strategic direction to address early childbearing and its ineffective teenage pregnancy reduction program.^17^ Key informants were clear that UN agencies played an important role in supporting the government to take action:

“ … it was actually our health colleague [at UNICEF] who initiated a discussion on coming up with a national strategy, but the strategy was to look at the adolescent health and wellbeing. They worked with UNFPA and WHO, and I think based on their discussion, they realized that opening up the discussion to look at the social norm of teenage pregnancy and child marriage – was when child protection came in.” *(Representative, UN agency)*

### To what extent did teenage pregnancy reduction become a national priority following the President’s declaration?

The extent to which teenage pregnancy reduction became a national priority in Sierra Leone was assessed using Schiffman’s framework for determining political prioritisation. With regard to the first criterion – that national political leaders publicly and privately express sustained concern for the issue – both political and local leaders continued to express their support for the NSRTP both publicly and privately during its implementation. Several key informants echoed that there was sustained support from all sectors, including political leaders:

“What facilitated the implementation of the program was commitment and making sure that everyone owns the program, it was not like just a government business, it was everybody’s business. People were invited from different sections which was great … political leaders including the president all expressed support for the issue following the declaration. You will hear from radio, people from ministries talking about teenage pregnancy.” *(Representative, NGO)*

“The Minister of Health and Sanitation by then provided a lot of political will for the implementation of programs geared towards reducing teenage pregnancy. And when you came down to the districts, you have the District Health Management Team, the District Council, politicians, chairpersons, so many officials and even officials from the Ministry of Social Welfare, Gender and Children’s Affairs, were all playing their role. Down to the villages and the paramount chiefs and section chiefs they were all providing support.” *(Representative, National Secretariat for the Reduction of Teenage Pregnancy)*

Additionally, and with regard to Schiffman’s second criterion for political prioritisation regarding authoritative decision-making processes, the GoSL took several steps toward the operationalisation of a plan to address teenage pregnancy. Together with development partners, the GoSL drafted the first NSRTP (2013–2015), wherein the Government acknowledged the problem and set out a framework for addressing it. The NSRTP also established mechanisms to oversee the strategy’s implementation, including the Multisectoral Coordination Committee to provide policy guidance and the Multisectoral Technical Committee to provide technical guidance, monitor implementation of activities and facilitate the sharing of technical information. Both committees were composed of government representatives and development partners. Additionally, the NSRTP mandated the establishment of the National Secretariat, which was set up and charged with the overall coordination of the initiative and with ensuring that teenage pregnancy remained on the national agenda. In addition to establishing these “mechanics” for implementation, the NSRTP included a two-track intervention plan involving, firstly, the review of policies and strategies to ensure the formal protection of adolescents’ rights and, secondly, the mobilisation of communities, the provision of services to young people, and the empowerment of women and girls.^8^

Further to the Government’s NSRTP commitment to improve the legal and policy environment, it enacted youth policies aimed at better protecting young people’s rights. In 2014, a national youth programme and an updated national youth policy were published.^29^ Despite the enactment of these youth-focused policies, not all actors were satisfied with the sustainability of the GoSL’s approach to teenage pregnancy reduction in the absence of a policy or law enshrining young people’s sexual and reproductive rights specifically. An NGO representative, for example, shared: “*I don’t think we have policies or laws in place to address teenage pregnancy in Sierra Leone. All I know is there are policies protecting girls like the Child Rights Act*”.

In relation to budgetary allocation – Schiffman’s third criterion – there were significant shortfalls from the beginning of the NRSTP. Whilst the government tried to synchronise the NSRTP with existing interventions that were already funded and implemented by a variety of ministries and development partners, there was a deficit of over US$23 million for the three years of implementation; this is acknowledged in the strategy document itself.^8^ Key informants agreed that the financial resources allocated for the strategy were, indeed, insufficient:

“There was a strategy … but the resources … to achieve the real goal of the secretariat [were] not there.” *(Representative, NGO)*

“In the MoHS, yes, government allocated and released funding, but for another ministry like the Ministry of Social Welfare [which was] the co-chair, there were limited resources … but the resources were not released directly for teenage pregnancy but were released to reduce violence against children and women in general.” *(Representative, UN agency)*

The key informants pointed to how challenging the resource limitations were for the NSRTP implementation. The National Secretariat, for example, struggled to be truly effective due to deficits in funding and human resources. As one representative of the MoHS put it, “*just creating it was not enough*”. The sense from key informants was that the lack of financial resources was at the heart of the Secretariat’s struggles; a representative of the Consortium on Teenage Pregnancy Prevention indicated that the National Secretariat had very little funding for its running costs, with most of the support coming from international agencies such as UNICEF, WHO and UNFPA. As a result, there was a sense amongst several key informants that the NSRTP became a “project” of international development partners:

“Funding is the first factor that obstructed the implementation of the first strategy. Political commitment was not one hundred percent, because if you are committed [and] you don’t give money [it] is difficult, then also the partners were looking at it as a vertical program on its own – So I believe that was also a challenge.” *(Representative, National Secretariat for the Reduction of Teenage Pregnancy)*

“From 2013 to 2015 there was no budget allocated [from the government]. If the government would have shown political commitment to the implementation from 2013 to 2015 there should have been funds. That particular strategy was 90% donor driven, UNICEF, Irish Aid and Save the Children.” *(Representative, National Secretariat for the Reduction of Teenage Pregnancy)*

### What factors facilitated or obstructed teenage pregnancy reduction remaining a political priority in Sierra Leone during the implementation of the first strategy (2013–2015)?

This study also considered factors that had a facilitative or hindering influence on teenage pregnancy reduction as a political priority during the implementation of the NSRTP. The literature and key informant data indicate that partnerships with NGOs and UN agencies were facilitating factors, ensuring that teenage pregnancy reduction remained on the agenda. On the other hand, the Ebola epidemic and sociocultural barriers presented challenges.

Non-governmental organisations such as Marie Stopes, Plan International, Restless Development and various United Nations agencies contributed to progress in relation to expected outputs of the NSRTP (see Figure 1). A UNICEF-BRAC programme to reduce pregnancy and child marriage set up and ran 200 adolescent development centres that have reached over 6000 girls aged 13–19 and provide financial literacy training, credit support for income generation activities and safe spaces for sharing. Other development partners also carried out initiatives aimed at empowering adolescents and young people to prevent and respond to teenage pregnancies; strategies used for this included the provision of microcredit to older adolescents, vocational training of out-of-school adolescents, community outreach on SGBV and human rights violations, engagement of boys through peer education networks, and strategic marketing of services.^22^

Moving to hindering factors, developments beyond the control of the government also affected teenage pregnancy reduction as a political priority. The Ebola epidemic in 2014 required a “diversion” of attention, including the redirection of the already-limited financial resources of partners and the government.^12,15,20^ Since the NSRTP was heavily supported by development partners, implementation experienced major setbacks as priorities shifted to addressing Ebola.^19^ This shift continued into the aftermath of the epidemic, when the focus was redirected toward economic recovery and restoring trust in government education and health systems, including the repurposing of Ebola Treatment Centers.^15^ One key informant summarised the impact as follows:

“The Ebola obstructed the strategy because when the Ebola broke out the government of Sierra Leone explicitly stated that all other [priorities] must be shifted towards the fight of the Ebola and all the efforts by health development partners should and must be geared [towards] the eradication of Ebola and when some people were insisting to do some teenage pregnancy work they were not even getting the attention of the DHMT [District Health Management Team].” *(Representative, National Secretariat for the Reduction of Teenage Pregnancy)*

The Ebola epidemic also had an impact on the availability and use of health services, evidenced in part by a spike in teenage pregnancies during the outbreak. By one estimate, 18,000 young women and girls became pregnant in Sierra Leone during the first cycle of the outbreak in 2014.^12^ In some regions of Sierra Leone, contraceptive services were completely unavailable during this time.^19^ The epidemic also affected the general public’s trust in and use of the health system due to a widespread belief that the system itself had fuelled the epidemic; this led to disengagement amongst the system’s primary users – women and children.^20^ Further, the economic slowdown and the deaths of parents and guardians during the Ebola outbreak led to an increase in young women’s engagement in transactional sex as a way to combat household poverty; this phenomenon is seen as having contributed to the increase in teenage pregnancy during the outbreak.^11^

Finally, the literature points to the effect that sociocultural barriers play in the implementation of programmes designed to promote the SRH of young people in the country. One author explained that, in Sierra Leone, policies centre on correcting girls’ “bad behaviour”, rather than on understanding the reasons behind girls’ decisions relating to sex and contraception.^11^ Further, the provision of sexuality education and contraception to adolescents still faces resistance from certain religious and cultural groups in the country.^2,11,26^

## Discussion

This study set out to answer a series of interrelated questions regarding the declaration made by the President of Sierra Leone in 2012 that put teenage pregnancy reduction on the country’s political agenda. The research attempted to shed light on the factors that led up to his decision, but also on the extent to which it led to political prioritisation. Whilst the quantity of peer-reviewed literature on the topic was limiting, this paper provides an historical overview based upon the relevant literature and the insights of those directly involved with the development and implementation of the first strategy to reduce teenage pregnancy in Sierra Leone.

In relation to the first research question, the findings indicate that the availability of data relating to teenage pregnancy and its consequences, as well as advocacy by international and national NGOs, were major motivating factors for the President’s initial declaration. Findings relating to the second question indicate that following the President’s commitment, the GoSL took steps to formalise it by drafting a strategy, establishing a National Secretariat and several committees, and adopting related strategies that fostered a supportive environment for implementation. At the same time, however, the GoSL did not allocate sufficient budget for the strategy’s implementation, relying instead on the support of international development partners for funding. Whilst there is evidence of some progress against all of the expected outputs of the strategy, the findings in relation to the third research question suggest that implementation stagnated due to resource constraints, the Ebola epidemic and sociocultural barriers relating to adolescent sexuality.

Overall, the findings show that public commitments made by political leaders and government officials – starting with President Koroma’s declaration in 2012 – kick-started efforts to reduce teenage pregnancy in Sierra Leone and, indeed, were sustained throughout the period of the NSRTP’s implementation. In turn, these publicly made commitments translated into the establishment of the systems and structures needed to reduce teenage pregnancy through the NSRTP and related mechanisms. However, several key informants lamented the short-term nature of these actions, instead calling for long-term policy – rather than time-bound strategy – for adolescent SRH. Whilst these first two of Schiffman’s three criteria for political prioritisation seem to have been fulfilled, the evidence does not support the same conclusion for his third criterion. There was near-unanimous consensus amongst the key informants and literature consulted for this study that the GoSL did not adequately resource the NSRTP or teenage pregnancy efforts more generally, though it should be noted that scarce indigenous resources were further depleted by the Ebola epidemic and its aftermath.

Whilst the “pathways” that countries take toward fulfilment of adolescent SRHR vary significantly, there are commonalities that can be observed. In particular, there are similarities in the motivation behind government action – namely, the impact of international policy and the availability and use of data to highlight the causes and consequences of teenage pregnancy. Policy responses at the global and regional levels have provided the impetus for country-level action in a variety of contexts. Beginning in 1994 when the International Conference on Population and Development Programme of Action positioned adolescent pregnancy as an issue deserving of global attention, international policy on the topic has “trickled down”, resulting in national responses such as the formulation of laws and policies, the provision of comprehensive sexuality education, the establishment of youth-friendly services and the increased contribution of domestic resources to SRH.^30^ In Sierra Leone, international organisations such as UNFPA, which were steeped in global conversations around teenage pregnancy, were instrumental in drawing attention to the need for a national level action, and those conversations were reinforced with a deluge of new, locally relevant data for advocates to utilise in highlighting the realities of young women. Indeed, in Sierra Leone, one statistic – that one every in three girls in Sierra Leone was pregnant before 18 – is credited with capturing the attention of a president.

This strategic use of data to highlight an issue of importance and to motivate government action can be seen in other contexts, too. In Chile, for example, it was the country’s high adolescent fertility rate that spurred the government into action with both international and national commitments to teenage pregnancy reduction. England is another example, where a goal to halve the teenage pregnancy rate in a decade ensured sustained political interest and provided a structured way of holding the government to account. Similarly, the Ethiopian government was motivated by maternal mortality reduction targets set pursuant to the MDGs.^30^

Since the ending of the NSRTP, Sierra Leone has developed and implemented several complementary strategies addressing adolescent SRH, including: the National Reproductive, Maternal, Child and Adolescent Health Strategy (2017–2021); the Basic Package of Essential Health Services (2015–2020); the National Strategy for the Reduction of Adolescent Pregnancy and Child Marriage strategy of 2018–2022; and the Education Sector Plan 2018–2020. Additionally, political leaders have made public commitments to reducing teenage pregnancy at the regional level (e.g. the African Union Campaign to End Child Marriage) and the international level (e.g. the Sustainable Development Goals and the Global Strategy for Women’s, Children’s and Adolescent Health (2016–2030)). Although this is beyond the scope of the paper, these commitments have seen results. Encouragingly, the most recent Multiple Indicator Cluster Survey report (2017) indicates that the adolescent birth rate for Sierra Leone fell to 101 nationally, down from 122 in the previous report from 2010.^31^ This exceeds the progress expected in the NSRTP, which set 110/1000 as the target adolescent fertility rate to achieve during the life of the strategy.^8^ Amongst young women aged 15–19 who are not in a union, 53.7% reported using a modern method of contraception.^31^ Further, modern contraceptive use has increased by 13% among married adolescents, from 1.2% in 2008 to 14.3% in 2019,^32,33^ and there has been an increase of 33% among sexually active unmarried adolescents from 21% in 2008 to 54% in 2013.^32,34^ Whilst it is not possible to attribute these changes directly to any one commitment or action, and whilst the limitations of the NSRTP are documented in this and other reports and studies, it may be said that the President’s commitment, the first NSRTP, and the programmes of government and development partners, contributed to tangible progress and positive trends over time.

Based on his experience with analysing factors that contributed to placing maternal mortality on the priority national agenda before the MDG era, Schiffman identified a number of factors working together, which he has placed in three categories – external influences, domestic advocacy and the national policy environment.^4^ In terms of external influence, he points to the promotion of norms and the provision of resources. In terms of domestic advocacy, he identifies political entrepreneurship, a cohesive policy community, focusing events, credible indicators and clear policy alternatives. Finally, in relation to the national policy environment, he points to political transitions and existing health priorities. When applied to teenage pregnancy reduction in Sierra Leone, it is clear that these same factors are at play. There were several external influences, including UN agencies, that had been advocating for long-term political commitment to teenage pregnancy reduction; this, together with domestic advocacy, was driven by the data that emerged around young people’s SRH. Of particular relevance in Sierra Leone is Schiffman’s third set of factors relating to national policy environments; he states that “political scientists have found that major political transitions and reforms such as democratisation and public sector decentralisation alter public priorities by giving new actors agenda-setting power, and by changing the processes by which public policies are made and implemented”.^4^ The centrality of teenage pregnancy to a myriad of development and health priorities, as well as the political transitions happening in the aftermath of the civil war, may have opened up political “space” for President Koroma to place teenage pregnancy reduction on the agenda.

The experience of Sierra Leone is one from which several lessons and recommendations for better responses to teenage pregnancy in low- and middle-income countries can be drawn, particularly for advocates. First, complementary advocacy efforts should take place at all levels – global, regional and national – ensuring that relevant data from the local context play a central role. Second, whilst there is value in a concerted focus on placing adolescent pregnancy on the political agenda, advocacy strategies should also include “asks” relating to the structures, systems, policies and, importantly, budget to support it. In other words, advocacy must continue beyond a public commitment or declaration into the implementation phase. Finally, as other political priorities emerge that may displace adolescent pregnancy – as was the case with the Ebola epidemic in Sierra Leone – advocates should adapt, ensuring that they respond to the needs of the hour.

## Limitations

There were a limited number of peer-reviewed publications related to the research questions available, though the research team gathered and reviewed every document available in the public arena that matched the inclusion criteria. As a result of this dearth of peer-reviewed publications, the paper relies primarily on the grey literature consulted and the 11 in-depth interviews with key informants. With regard to the key informant interviews, however, every effort was made to ensure representation from all stakeholder groups – government, NGOs and UN officials – and to ensure that a “saturation point” was reached. That said, no young people were involved in the drafting of this paper, although this was because they did not have a substantive role in the development of the NSRTP in Sierra Leone. Another limitation is that, although Schiffman’s framework was used to inspire the paper and his criteria for political prioritisation were used in analysing the data in relation to the second research question, his method of process-tracing was not used. Finally, we reiterate that the intention of the paper is to provide insight to countries with similar contexts wishing to implement policies or strategies on adolescent and young people’s SRH, rather than to comment fully on whether or not the NSRTP achieved its intended results through implementation.

## Conclusion

Public declarations made by political leaders can often represent the beginning of a government’s commitment to an issue. In the case of Sierra Leone, this study demonstrates how advocacy from both international and national NGOs and the strategic use of up-to-date data on adolescent SRH drew the President’s attention to the issue of teenage pregnancy in 2012. His commitment amidst the myriad of development issues facing the country in the aftermath of its civil war resulted in the first teenage pregnancy reduction strategy and the establishment of a national secretariat. Whilst the political commitment to teenage pregnancy and commensurate policy actions remained through the tenure of the NSRTP, the financial resource deficits proved a significant challenge to ensuring that the intended outputs were met. Further, the Ebola epidemic shifted health and development efforts away from teenage pregnancy in 2014 and beyond. Since the conclusion of the first strategy, Sierra Leone has continued to prioritise the topic of teenage pregnancy in health policy and has seen a reduction in its overall adolescent fertility rate during the past decade. For advocates wishing to put teenage pregnancy on the political agenda, whilst also ensuring that it remains a priority, many lessons can be learnt from the experience of Sierra Leone.

## References

[1] United Nations. The millennium development goals report; 2015. [cited 2020 Jan 8]. Available from: https://www.un.org/millenniumgoals/2015_MDG_Report/pdf/MDG%202015%20rev%20(July%201).pdf

[2] Bransky R, Enoch J, Long C. Child marriage in Sierra Leone and Guinea: cultural roots and girl centred solutions. Freetown: Purposeful Productions; 2017.

[3] Chandra-Mouli V, Ferguson J, Plesons M, et al. The Political, Research, Programmatic, and Social Responses to Adolescent Sexual and Reproductive Health and Rights in the 25 Years since the International Conference on Population and Development. J Adolesc Health. 2019;65(6):S16–S40. doi: 10.1016/j.jadohealth.2019.09.011 31761001

[4] Shiffman J. Generating Political Priority for Maternal Mortality Reduction in Five Developing Countries. Am J Public Health. 2007;97(5):796–803. doi: 10.2105/AJPH.2006.095455 17395848 PMC1854881

[5] Statistics for Sierra Leone (SSL). Population and Housing Census – summary of final results; 2015. [cited 2020 Jan 8]. Available from: https://fenam.online/assets/pdf/2015_Population_And_Housing_Census%20_21-12-16.pdf

[6] Statistics Sierra Leone (SSL). Sierra Leone Integrated Household Survey Report 2018; 2019. [cited 2020 Jan 8]. Available from: https://www.statistics.sl/images/StatisticsSL/Documents/SLIHS2018/SLIHS_2018_New/sierra_leone_integrated_household_survey2018_report.pdf

[7] Stark L, Tan TM, Muldoon KA, et al. Family structure and sexual and reproductive health outcomes among adolescents in rural Sierra Leone. Glob Public Health. 2015;11(3):309–321. doi: 10.1080/17441692.2015.1031155 25880353

[8] Government of Sierra Leone (GoSL). Let girls be girls, not mothers! National Strategy for the Reduction of Teenage Pregnancy (2013–2015); 2013. [cited 2020 Jan 8]. Available from: https://hivhealthclearinghouse.unesco.org/sites/default/files/resources/Sierra_Leone_National_Strategy_for_the_Reduction_of_Teenage_Pregnancy.pdf

[9] Statistics Sierra Leone (SSL) & UNICEF-Sierra Leone. Sierra Leone Multiple Indicator Cluster Survey 2010, Final Report. Freetown: Author; 2011. [cited 2020 Jan 10]. Available from: https://reliefweb.int/sites/reliefweb.int/files/resources/MICS4_SierraLeone_2010_FinalReport.pdf

[10] Akara A. Lessons from girls’ empowerment and leadership programs in Sierra Leone, Liberia and Ghana. Int Fem J Politics. 2015;17(2):339–344. doi: 10.1080/14616742.2015.1014257

[11] Bash-Taqi R. A review of the policy environment in Sierra Leone to inform its level of readiness for the implementation of the adolescent health element of the WHO global strategy for women’s children’s and adolescents’ health; 2018. Unpublished manuscript.

[12] Bruce J. The difficulties of ‘living while girl’. J Virus Erad. 2016;2(3):177–182. doi: 10.1016/S2055-6640(20)30462-3 27482459 PMC4967971

[13] Coinco E. A Glimpse into the World of Teenage Pregnancy in Sierra Leone. Freetown: UNICEF; 2010. [cited 2020 Jan 8]. Available from: https://www.humanitarianresponse.info/sites/www.humanitarianresponse.info/files/assessments/teenage_pregnancy_report_unicef_final_aug_2010_2.pdf

[14] De Koning K, Jalloh-Vos H, Kok M. Realities of teenage pregnancy in Sierra Leone. Amsterdam: KIT; 2013. [cited 2020 Jan 8]. Available from: https://www.kit.nl/wp-content/uploads/2018/08/2086_sierra-leone_web.pdf

[15] Denney L, Gordon R, Ibrahim A. Teenage Pregnancy after Ebola in Sierra Leone: mapping responses, gaps and ongoing challenges. London: Secure Livelihoods Research Consortium: Overseas Development Institute (ODI); 2015. [cited 2020 Jan 8]. Available from: https://securelivelihoods.org/wp-content/uploads/Teenage-Pregnancies-after-Ebola-in-SierraLeone_-Mapping-responses-gaps-and-ongoing-challenges.pdf

[16] Denney L, Gordon R, Kamara A, et al. Change the context not the girls: Improving efforts to reduce teenage pregnancy in Sierra Leone. SLRC report. London: ODI; 2016.

[17] Diarra M, Kouame K, Kaikai F. Children Bearing Children: the Determinants and Consequences of Teenage Pregnancy and Motherhood in Sierra Leone. Sierra Leone: UNFPA; 2011. [cited 2020 Jan 9]. Available from: https://sierraleone.unfpa.org/sites/default/files/pub-pdf/UNFPA-Children_Bearing_Children-SL.pdf

[18] Dunlop J, Penzhorn N. Adolescent girl club in Sierra Leone tackles teenage pregnancy and engenders independence. UNICEF; 2014. Available from: https://www.unicef.org/protection/sierraleone_74277.html

[19] Ebola Deeply. Ebola and women: Chernor Bah on the impact on girls in Sierra Leone; 2014. [updated 2020 Jul 20; cited 2020 Jan 8]. Available from: http://archive.eboladeeply.org/articles/2014/11/6494/ebola-women-chernor-bah-impact-girls-sierra-leone/

[20] Elston JWT, Moosa AJ, Moses F, et al. Impact of the Ebola outbreak on health systems and population health in Sierra Leone. J Public Health (Bangkok). 2015;38(4):673–678. doi: 10.1093/pubmed/fdv158 28158472

[21] Farzaneh N. An Evaluation of Teenage Pregnancy Pilot Projects in Sierra Leone. Freetown: UNICEF; 2013.

[22] Kosia AB. A Close Look at Teenage Pregnancy and its Intervention Strategies in Sierra Leone [Unpublished capstone submitted in partial fulfillment for the degree of Masters of Public Health]. Simon Fraser University, Burnaby, Canada; 2015.

[23] Republic of Sierra Leone. Assessment and contributing factors to teenage pregnancy in Sierra Leone. Sierra Leone: Ministry of Health, National Secretariat for the Reduction of Teenage Pregnancy (NSRTP); 2016.

[24] Restless Development. Young people in Sierra Leone today, chapter four: sexual and reproductive health. Freetown: Restless Development; 2012.

[25] Runsewe-Abiodum T, Bondi SF. Teenage pregnancy and implications on child survival amongst mothers attending a clinic in the east-end, Freetown, Sierra Leone. Open J Pediatr. 2013;3:294–299. doi: 10.4236/ojped.2013.34053

[26] Shirley A, Lilley S, Stark L, et al. Preventing Teenage Pregnancy in Sierra Leone. Impact Evaluation Baseline Report. London: Interagency Learning Initiative on Community-Based Child Protection Mechanisms and Child Protection Systems; 2014. [cited 2020 Jan 9]. Available from: http://www.cpcnetwork.org/wp-content/uploads/2014/12/311014-Sierra-Leone-IS-Baseline-Report.pdf

[27] Stark L, Macfarlane M, King D, et al. A community-driven approach to reducing teenage pregnancy in Sierra Leone: midline evaluation brief. London: Save the Children; 2014.

[28] Steifel. How did Ebola Impact Maternal and Child Health in Liberia and Sierra Leone? Washington (DC): Center for Strategic and International Studies; 2015.

[29] Ministry of Youth Affairs and National Youth Commission. Sierra Leone. A Blueprint for Youth Development: Sierra Leone’s National Youth Programme 2014–2018; 2014. [cited 2020 Aug 14]. Available from: https://erc.undp.org/evaluation/managementresponses/keyaction/documents/download/672

[30] Chandra-Mouli V, Plesons M, Hadley A, et al. Lessons learned from national government-led efforts to reduce adolescent pregnancy in Chile, England and Ethiopia. Early Child Matters. 2019;128:50–56. Available from https://earlychildhoodmatters.online/2019/lessons-learned-from-national-government-led-efforts-to-reduce-adolescent-pregnancy-in-chile-england-and-ethiopia/

[31] Statistics Sierra Leone (SSL). Sierra Leone Multiple Indicator Cluster Survey 2017, Survey Findings Report. Freetown: Statistics Sierra Leone; 2018. [cited 2020 Jan 10]. Available from: https://www.statistics.sl/images/StatisticsSL/Documents/sierra_leone_mics6_2017_report.pdf

[32] Statistics Sierra Leone and ICF Macro. Sierra Leone Demographic and Health Survey 2008. Calverton: SSL and ICF Macro; 2009.

[33] Statistics Sierra Leone (Stats SL) and ICF. Sierra Leone Demographic and Health Survey 2019: key indicators. Freetown: Stats SL and ICF; 2019.

[34] Statistics Sierra Leone (SSL) and ICF International. Sierra Leone Demographic and Health Survey 2013. Freetown: SSL and ICF International; 2014.

